# A randomized open-label study of guideline-driven antiemetic therapy versus single agent antiemetic therapy in patients with advanced cancer and nausea not related to anticancer treatment

**DOI:** 10.1186/s12885-018-4404-8

**Published:** 2018-05-02

**Authors:** Janet Hardy, Helen Skerman, Paul Glare, Jennifer Philip, Peter Hudson, Geoffrey Mitchell, Peter Martin, Odette Spruyt, David Currow, Patsy Yates

**Affiliations:** 1 Mater Misericordiae Limited, Mater Research - University of Queensland, Raymond Terrace, Brisbane, QLD 4101 Australia; 2 Institute of Health and Biomedical Innovation (IHBI), Faculty of Health, Queensland University of Technology, Level 7, Q Block, Kelvin Grove Campus, Brisbane, QLD 4059 Australia; 3 Pain Management Research Institute, Royal North Shore Hospital, St Leonards, NSW 2065 Australia; 4 St Vincent’s Hospital and the University of Melbourne, Fitzroy, VIC 3065 Australia; 5 St Vincent’s Hospital & Collaborative Centre of The University of Melbourne, PO Box 2900, Fitzroy, VIC 3065 Australia; 6 University of Melbourne, Melbourne, Australia; 7 School of Medicine, The University of Queensland, QLD, Brisbane, 4072 Australia; 8 Barwon Health McKellar Centre, 45-95 Ballarat Rd, North Geelong, VIC 3215 Australia; 9 Peter MacCallum Cancer Centre, 305 Grattan Street, Melbourne, VIC 3000 Australia; 10 Faculty of Health, University of Technology, Sydney, Australia; 11 Queensland University of Technology, VIC Park Rd, Kelvin Grove, QLD 4059 Australia; 12 Department Palliative and Supportive Care, Mater Health Services, Raymond Terrace, South Brisbane, QLD 4101 Australia

**Keywords:** Nausea, Vomiting, Palliative care, Antiemetic, Guidelines

## Abstract

**Background:**

Nausea/vomiting (N/V) not related to anti-cancer treatment is common in patients with advanced cancer. The standard approach to management is to define a dominant cause, and treat with an antiemetic selected through pathophysiologic knowledge of emetic pathways. High rates of N/V control have been reported using both etiology-based guideline-driven antiemetic regimens and an empiric approach using single agents in uncontrolled studies. These different approaches had never been formally compared.

**Methods:**

This randomized, prospective, open label, dose-escalating study used readily available antiemetics in accordance with etiology-based guidelines or single agent therapy with haloperidol. Participants had a baseline average nausea score of ≥3/10. Response was defined as a ≥ 2/10 point reduction on a numerical rating scale of average nausea score with a final score < 3/10 at 72 h.

**Results:**

Nausea scores and distress from nausea improved over time in the majority of the 185 patients randomized. For those who completed each treatment day, a greater response rate was seen in the guideline arm than the single agent arm at 24 h (49% vs 32%; *p* = 0.02), but not at 48 or 72 h. Response rates at 72 h in the intention to treat analysis were 49 and 53% respectively, with no significant difference between arms (0·04; 95% CI: -0·11, 0·19; *p* = 0·59). Over 80% of all participants reported an improved global impression of change. There were few adverse events worse than baseline in either arm.

**Conclusion:**

An etiology-based, guideline-directed approach to antiemetic therapy may offer more rapid benefit, but is no better than single agent treatment with haloperidol at 72 h.

**Clinical trial registration:**

Australian New Zealand Clinical Trials Registry: ANZCTRN12610000481077.

## Background

Nausea and vomiting (N/V) in patients with advanced cancer is common, chronic and distressing with prevalence rates of up to 70% [1, 2]. In one study, 25% of people admitted to a palliative care unit had nausea ratings of > 50 on a 0–100 visual analogue scale [3]. The negative influence on activities of daily living is significant [4, 5]. N/V is often associated with other symptoms such as pain and reflux within specific clusters [6].

There are multiple potential causes including organ failure, central nervous system disease, drug therapy and gastrointestinal obstruction/pathology, often with several contributory factors in any one patient [7]. Management involves treatment of the underlying cause(s), supportive care measures (eg removal of bad odors, control of anxiety) and the delivery of antiemetics.

Current teaching when treating nausea is to define the predominant cause and combine knowledge of the pathophysiology of the vomiting process with the neuropharmacology of emetic pathways to determine which receptors and neurotransmitters are best to target therapeutically. The antiemetic medications known to be most active at those receptors can then be prescribed [8]. A specific cause is said to be identifiable in up to 90% of patients [1]. Several audits and uncontrolled studies have tested this etiology-based approach and have demonstrated high rates of nausea control [9–12]. Others have used single antiemetic agents for all patients, irrespective of the cause and have demonstrated similarly high rates of control [1].

The two different approaches have not previously been tested against each other. In this study, the effectiveness of a guideline-directed etiology-based approach to antiemetic management was compared to single agent (empirical) management. The former was undertaken using a clinical practice guideline, previously developed according to best evidence of effect at the time [9]. Haloperidol was selected for the single agent arm, as it is recommended as standard therapy for nausea in palliative care practice [7, 8].

## Methods

### Study design

This open label, randomized controlled parallel arm trial was undertaken by the Palliative Care Clinical Studies Collaborative (PaCCSC) in 11 sites across Australia. The study was approved by Human Research Ethics Committees covering all sites (Alfred Hospital (Victoria lead), Hunter New England (NSW lead), Mater Health Services, Southern Adelaide, St. Vincent’s Health & Aged Care and Queensland University of Technology).

### Patients

Participants > 18 years, had a diagnosis of cancer and nausea with an average score of ≥3 on an 11 point (0–10) NRS. They were not currently receiving antiemetics or had received inappropriate antiemetics (as defined by the antiemetic guidelines).

Patients were excluded if they: had a short term iatrogenic or reversible cause of nausea for which there was high level evidence that a specific antiemetic or intervention was indicated (e.g., raised intracranial pressure or acute chemo-radiotherapy induced nausea), were likely to undergo any procedure with the potential to affect nausea in the two days prior, or during the study period, had a definite contraindication to any of the study medications, a change in glucocorticoid dose within 48 h, or poor performance status (that would have rendered the participant unable to complete study requirements).

### Interventions

Investigators received formal training regarding the different mechanisms of nausea and vomiting and how to determine a primary cause. All potential causes were recorded and a dominant mechanism defined if possible.

Randomization schedules were computer-generated for each site at an independent central registry. Schedules for each site were allocated in a 1:1 ratio in randomly allocated blocks of two or four. Schedules held by the central registry were sent to each site in opaque sealed envelopes numbered in sequence. On notification of an eligible patient, the research coordinator at each site opened the next numbered envelope, allocated the patient to the guideline treatment or single therapy arm, and notified research staff and treating clinicians of the treatment group allocation. It was not possible to blind treatment in view of the number of medications utilized and the complexities of dose escalation.

Those allocated to the guideline treatment group received antiemetic therapy based on the etiology-based clinical practice guidelines (CPGs) developed from a systematic review of the efficacy of antiemetics in patients with far-advanced cancer by Glare et al. [9]. The CPGs were updated and modified by consensus using an expert panel following review of the recent literature. The CPGs (Table 1) specify common mechanisms of nausea (categories A-G) and for each mechanism, recommend first line treatment, with subsequent lines of treatment in a step-wise fashion, every 24 h, if nausea remained uncontrolled. For multiple contributing factors, investigators were instructed to treat according to the primary or dominant contributing factor. The CPGs were adapted to include recommended treatment options for patients in whom a primary cause could not be determined (category H). The oral route was preferred for all study medicines, but parenteral routes were allowed for those unable to take oral medications.

**Table 1:** Clinical practice guidelines for the management of nausea

Dominant cause	Treatment Step 1	Treatment Step 2	Treatment Step 3
A: Central/chemoreceptor trigger zone (CTZ) stimulation	Prochlorperazine 5 mg tds po or 25 mg PR then 5 mg tds po or 12.5 mg bd IM/iv	Haloperidol 1.5 mg/24 h po or sc	Haloperidol 3 mg/24 h po or sc
B: Central nervous system (CNS) disease	Dexamethasone 8 mg/24 h po/sc/iv	Dexamethasone 12 mg/24 h po/sc/iv	Dexamethasone 16 mg/24 h po/sc/iv
C: Vestibular involvement	Prochlorperazine 5 mg tds po or 25 mg PR then 5 mg tds po or 12.5 mg bd IM/iv	Prochlorperazine 10 mg tds po or 25 mg PR then 10 mg tds po or 12.5 mg tds IM/iv	Promethazine 25 mg tds po or 12.5 mg sc then 10 mg tds po
D: Gastric stasis	Metoclopramide 10 mg qid po/sc/iv	Metoclopramide 10 mg Q4h po/sc/iv	Metoclopramide 10 mg Q4h po/sc/iv Dexamethasone 8 mg/24 h po/sc/iv
E: Ileus	Metoclopramide 10 mg qid po/sc/iv	Metoclopramide 10 mg Q4h po/sc/iv	Metoclopramide 10 mg Q4h po/sc/iv Dexamethasone 8 mg/24 h po/sc/iv
F: Mechanical obstruction	Haloperidol 1.5 mg/24 h po/sc Dexamethasone 8 mg/24 h po/sc/v	Haloperidol 3 mg/24 h po/sc Dexamethasone 8 mg/24 h po/sc/iv	Haloperidol 3 mg/24 h po/sc Dexamethasone 8 mg/24 h po/sc/iv Hyoscine butylbromide 80 mg/24 h sc *or* Ranitidine 200 mg/24 h sc
G: Gastritis	Metoclopramide 10 mg qid po/sc/iv PPI min dose	Metoclopramide 10 mg qid po/sc/iv PPI max dose	Metoclopramide 10 mg Q4h po/sc/iv PPI max dose
H: Cause undetermined (or multifactorial)	Metoclopramide 10 mg qid po/sc/iv	Metoclopramide 10 mg qid po/sc/iv Haloperidol 1.5 mg/24 h po/sc	Metoclopramide 10 mg Q4h po/sc/iv Haloperidol 3 mg/24 h po/sc

*Po* by mouth, *PR* per rectum, *sc* subcutaneous, *iv* intravenous, *IM* intramuscular, *bd* twice daily, *tds* three times daily, *qid* four times daily, *Q4h* four hourly, *PPI* proton pump inhibitor, *Min* minimum, *Max* maximum

Participants allocated to the single agent group received haloperidol (1.0 mg/24 h) orally or parenterally (subcutaneous or intravenous). In patients with uncontrolled nausea, the dose was increased in a step-wise fashion every 24 h to 2 mg and then to a maximum of 3 mg/24 h.

Metoclopramide 10 mg was charted as a rescue antiemetic to be given 4 hourly as required (prn), except in those arms where metoclopramide was already being delivered 4 hourly in which case haloperidol (0·5 mg prn to 6 hourly) was used for breakthrough.

Patients who did not meet the response definition at 24 h proceeded to second and third line antiemetic therapy according to the CPGs. Patients who met the response definition at 24 h remained on the same antiemetic regimen. At 72 h, participants whose nausea had been controlled remained on the effective medication and dose level. Patients with refractory nausea (final score ≥ 3/10) were managed at the discretion of the treating clinician.

### Outcome measures

Assessments of nausea severity and distress were undertaken daily. Other measures were: performance status [13], symptom burden [14], quality of life [15], co-morbidities [16], and toxicity [17, 18].

A response was defined as at least a 2-point reduction in average nausea score and a score < 3 for average nausea over the preceding 24 h, measured at 72 h on an 11-point numerical rating scale (NRS).

The primary outcome measure was a response at 72 h (end day 3). Secondary outcomes measured at 72 h included: average, best and worst nausea scores, nausea distress, the number of patients treated at each dose level, rescue doses delivered, episodes of vomiting, global impression of change and adverse events.

### Statistical analysis

The primary hypothesis of no difference in response rates at 72 h was tested using chi-square tests of differences between treatments. Assuming a response rate of 75% in the guideline arm and 50% in the single agent arm, a minimum of 150 participants (75 per group) who completed 72 h of treatment was deemed adequate to detect an absolute and clinically relevant difference in response rates of 25% between single agent and guideline therapy, with 90% power, assuming a two-tailed Type 1 error of 5% and a simple random sampling scheme.

In the primary intention-to-treat (ITT) analysis, randomized patients who withdrew for any reason were classified as ‘non-responders’. A secondary analysis was conducted as a ‘*per protocol*’ analysis of patients who completed 72 h of trial medication. Descriptive statistics were generated from the patient’s demographic and clinical characteristics. Chi-square analysis was used to detect any differences between treatment groups at baseline.

In secondary cross-sectional analyses, each endpoint was considered separately. Regression modeling of nausea scores over time and the impact of the intervention on these trends were conducted under the assumption of ‘missing at random’, implementing a linear mixed models approach to adjust for potential confounding and ensure that individuals with missing data over time may be included as far as their data permit. Results are expressed as prevalence rates, median and range, mean and standard deviation, depending on the data type and analysis.

## Results

**Fig. 1:**
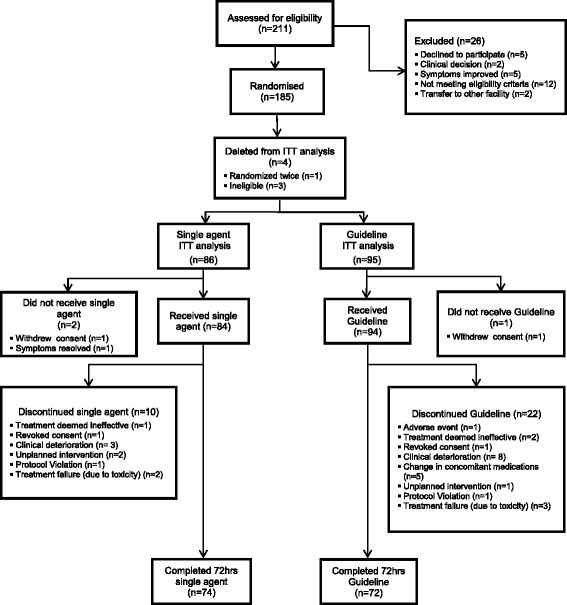
Participant flow

Of the 211 potential participants assessed for eligibility, 185 patients were randomized between October 2010 and April 2014. Four patients (all randomized to single agent) were subsequently deleted from the analysis: one patient was randomized twice and three patients were subsequently proven to be ineligible. The intention-to-treat (ITT) sample comprised 86 patients assigned to single agent treatment and 95 to the guideline group. Patient flow is presented in Fig. 1. At 72 h, 74 patients had completed single agent treatment and 72 guideline treatment, giving an attrition rate of 21% (35/181).

Randomization achieved two groups that were similar with respect to baseline characteristics (Table 2). The average and worst nausea scores at baseline in both arms was around 5/10 and 7/10. Nausea distress was reflected by a mean nausea distress score of around 5/10 in both arms. The majority of participants (85%) had received anti-emetics in the week prior to study. Nausea was considered to be multi-factorial in origin in 79% of participants randomized to single agent and 71% to guideline therapy. A dominant cause for N/V (most commonly central/CTZ stimulation and gastric stasis) could be determined in 47% of single agent and 60% of guideline participants.

**Table 2:** Patients’ demographic and medical characteristics by treatment arm

	Single agent (*n* = 86)				Guideline Therapy (*n* = 95)			
Characteristic	No.	%	Mean	SD	No.	%	Mean	SD
Age (years)			69.3	14.3			68.1	13.0
Male gender	23	27.4			34	35.8		
Place of care								
Inpatient palliative care	29	34.5			36	37.9		
Hospital general ward	28	33.3			37	38.9		
Private home	27	32.1			22	23.2		
Primary Cancer Diagnosis								
Breast	8	9.3			15	15.8		
Lung	9	10.5			12	12.6		
Colorectal	12	14.0			9	9.5		
Gynaecologic	14	16.3			12	12.6		
Gastrointestinal	4	4.7			6	6.3		
Pancreas	6	7.0			5	5.3		
Prostate	5	5.8			11	11.6		
Other	23	26.7			19	20.0		
Unknown	5	5.8			6	6.3		
Performance status^a^ (0–100)								
Median (Interquartile range)	60 (50–70)				60 (50–70)			
Quality of life^b^ (1–7)			3.0	1.3			3.0	1.3
Symptom burden^c^ (0–90)			37.3	16.1			39.0	13.7
Charlson Comorbidity Index			6.1	2.4			6.6	2.1
Vomited in last 24 h	28				30	31.6		
(Yes)		32.6						
Number of vomiting episodes	0				0	(0–10)		
Median (range)		(0–5)						
Duration of current nausea	13				20	21.1		
< 1 week	18				26	27.4		
1 up to 2 weeks	18	15.5			21	22.1		
2 up to 4 weeks	15	21.4			13	13.7		
1 up to 2 months	20	21.4			15	15.8		
≥ 2 months		17.9						
		23.8						
Nausea score (0–10)								
Worst			7.6	1.8			7.4	1.9
Best			2.1	1.8			1.8	2.0
Average			5.2	1.4			5.0	1.5
Distress			4.9	3.0			5.2	3.2
Nausea Interference^d^ (0–100)			43.6	27.3			43.8	26.0
Nausea - multi-factorial	68	79.1			67	70.5		
Dominant cause								
Undetermined	46	53.5			38	40.0		
Central/CTZ^e^ stimulation	16	18.6			27	28.4		
Gastric stasis	8	9.3			12	12.6		
Other	16	18.6			18	18.9		
Adverse event^f^								
Fatigue	63	73.3			74	77.9		
Anticholinergic effects	48	55.8			63	66.3		
Gastrointestinal upset	51	59.3			57	60.0		
Anorexia	50	58.1			57	60.0		
Drowsiness	42	48.8			53	55.8		
Dizziness	17	19.8			17	17.9		
Hyper/hypotension	12	14.0			14	14.7		
Restlessness	4	4.7			5	5.3		
Extrapyramidal reactions	1	1.0			1	1.0		
Incoordination	0	0.0			4	4.2		

^a^Australian-modified Karnofsky performance status scale

^b^EORTC-QLQ-C15-PAL

^c^Edmonton Symptom Assessment Scale

^d^Nausea Interference Scale

^e^Chemo Receptor Trigger Zone

^f^Any grade

In the primary (ITT) analysis, the response to treatment at 72 h was 53% (46/86) in the single agent arm and 49% (47/95) in the guideline therapy arm with no difference between groups (0.04; 95% CI: -0.11, 0.19; *p* = 0.59). In the *per protocol* analysis, there was no difference in response rates; (62% (46/74) single agent and 65% (47/72) guideline therapy) (− 0.03; 95% CI: -0.19, 0.13; *p* = 0.70). Similarly, there was no difference in response rates between arms (ITT) using ‘worst’ nausea. For those who completed each treatment day, greater response rates were seen in the guideline arm than the single agent arm at 24 h (49% vs 32%, *p* = 0.02), but not at 48 (69% vs 59%, *p* = 0.17) and 72 h (65% vs (62%, *p* = 0.70).

**Fig. 2:**
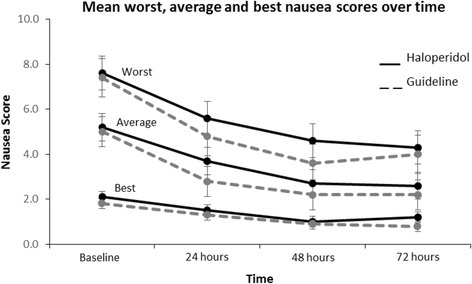
Mean score - worst, average and best nausea by treatment over time

Multivariate analyses of nausea scores as continuous variables were conducted to investigate the effects of time, group and time*group interaction. From the trajectory of mean nausea scores (Fig. 2), average, best and worst nausea scores all improved over time (*p* < .001). Only mean scores of average nausea differed significantly between treatment arms.

After treatment completion (72 h), patients in both arms were less distressed by nausea. Mean distress scores for single agent were 2.5 (95% CI, 1.9–3.2) compared to baseline 4.9 (95% CI: 4.2, 5.5) and 2.3 (95% CI: 1.6, 3.0) compared baseline 5.2 (95% CI: 4.6, 5.8) for guideline therapy.

The percentage of participants treated at each dose level over time is shown in Table 3. The majority of participants completed the study on steps 1 or 2. Approximately one-third of all participants were given rescue antiemetics each day with a greater use of rescue medications in the single agent arm at 48 h (43% vs 22%, *p* = 0.003) but not at 24 or 72 h (42% vs 33%, *p* = 0.25 and 35% vs 27%, *p* = 0.31).

**Table 3:** Proportion of patients in each treatment arm by current dose step at each time

Time	Dose step	Single agent (haloperidol)		Guideline therapy		Total		Chi-square, *p*-value
		N	n (%)	N	n (%)	N	n (%)	
24 h	Step 1	82	82 (100)	92	92(100)	174	174 (100)	
48 h^a^	Step 1		28 (37.3)		45 (54.2)		74 (46.5)	
	Step 2	75	47 (62.7)	83	38 (45.8)	158	85 (53.5)	Χ^2^ = 4.5, *p* = 0.03
72 h	Step 1	75	23 (30.7)	74	36 (48.4)	149	59 (39.6)	
	Step 2		30 (40.0)		25 (33.8)		55 (36.9)	Χ^2^ = 5.6,
	Step 3		22 (29.3)		13 (17.6)		35 (23.5)	*p* = 0.06

^a^One patient on Step 1 escalated to Step 3 due to rescue medications given

The percentage of participants reporting ≥1 episode of vomiting/day decreased from around 33% at baseline to 17% at 72 h in both treatment arms. At 72 h, compared to 24 h, the proportion of patients reporting improvement in global impression of change (GIC) increased from 64 to 86% (single agent) and from 70 to 82% (guideline therapy). In a longitudinal analysis, GIC improved over time (*p* < ·001) with no difference between treatment arms.

Adverse events were common at baseline with over 50% of all participants reporting fatigue, gastrointestinal upset, anorexia or anticholinergic effects (dry eyes, dry mouth, tremor) (Table 2). Treatment related adverse events are shown in Table 4.

**Table 4:** Number of Adverse Events graded worse at 72 h than at baseline

Adverse Event^a^	Single agent (haloperidol)	Guideline Therapy
Drowsiness	14	11
Fatigue	8	12
Anticholinergic effects	12	8
Gastrointestinal upset	10	10
Anorexia	4	5
Restlessness	3	3
Hyper/hypotension	4	3
Dizziness	3	5
Incoordination	3	4
Restless	3	3
Extrapyramidal reactions	0	1

^a^Any grade

## Discussion

Contrary to current teaching [8], we have not shown aetiology-based guideline directed therapy aimed at the presumptive cause of nausea to be superior to the regular administration of a single agent (haloperidol) at 72 h. Moreover, it has shown that N/V not acutely related to anti-cancer therapy can be controlled relatively rapidly in the majority of patients with cancer, at least in the short term, using currently available inexpensive medications regularly at recommended dosing levels.

The guidelines determined a specific treatment pathway for these patients based on best available evidence of antiemetic efficacy, using freely available antiemetics approved for this indication. The evidence to guide antiemetic use in this scenario is poor, with no high quality randomized controlled trials of individual drugs identified in the most recent review that recommends metoclopramide as the single drug of choice, with haloperidol listed as an appropriate second-line agents [19]. Haloperidol was selected as the single agent for this study as it is a relatively broad spectrum antiemetic used commonly in supportive and palliative care [7] and because metoclopramide was often the drug of choice within the guideline. Doses chosen for initiating and titrating therapy were determined by consensus as robust dose-ranging studies were not available.

Compliance with the allocated medication regimen ranged from 83 to 93% with a tendency towards greater compliance in the guideline arm. This was a “real world” study meant to mirror everyday practice rather than a rigid dose escalation trial. Similarly, the use of both oral and parenteral formulations reflects patient need in advanced disease.

The guideline approach resulted in superior nausea control at 24 h and in the use of rescue antiemetics at 48 h, but the benefit was not sustained at 72 h. This is important when aiming for a rapid improvement in symptom control and quality of life in patients with advanced disease.

The medications were well tolerated. There was a single report of an extra-pyramidal reaction. This low rate is consistent with reports in other palliative care populations [20].

It is often not possible nor appropriate to undertake complex investigations to look for an exact cause of nausea in patients with advanced disease [8, 21]. Those with nausea of known cause for which there is a clear evidence-based treatment (such as 5HT3 antagonists for chemo/radiotherapy induced vomiting), were excluded. A primary cause could only be defined in 54% of all participants which is lower than that reported in uncontrolled studies assessing guideline therapy and limits the usefulness of an etiology-based approach [1, 9]. The majority of participants in whom a cause could be determined were thought to have nausea related to a central cause, often medication related.

This study focused on nausea, whereas most other studies in cancer patients have used complete control of emesis as the primary end-point in the setting of chemotherapy-induced nausea and vomiting [22]. The need to control delayed nausea following chemotherapy has been recognized [23] and of “freedom from clinically significant nausea” (a nausea score of < 3 on an 11-point scale), has been used as an end-point [24]. The response definition used in this study was extrapolated from the pain literature in which a 2-point reduction on an 11-point NRS is considered clinically significant [25]. A final score within the “severe nausea” category is unlikely to reflect a clinically meaningful response hence our requirement for a final score of ≤3/10.

Guideline therapy is more difficult to institute. The sample size was calculated on the premise that a 25% improvement over single agent therapy would be clinically relevant and necessary in order to support the practice.

It was not possible to blind this study in view of the multiple medications involved and the complexity of the dose prescription and escalation process. Similarly, this was a short 3-day study as there is an urgency to relieve symptoms in this population group. Whether the results could be duplicated over a longer time period is unknown. There was some overlap in the guidelines with several arms targeting gastrointestinal causes of N/V. Other guidelines have relied more on investigations to determine a likely cause of nausea before deciding on appropriate treatment [26]. The primary aim of this study was to test an etiology-based guideline approach to antiemetic control. This resulted in the potential limitation of testing “like with like” in that haloperidol was also a part of the CPG. This could have contributed to the null result. Similarly, several of the antiemetics used are dopamine receptor antagonists with a similar mechanism of action as haloperidol.

## Conclusion

We have been unable to show that a mechanistic approach to N/V is superior to an empirical approach using a single agent given at adequate dose regularly in a dose-escalated manner. Moreover, N/V is often multifactorial or the primary cause cannot be determined. The response rate in this study was high in both arms suggesting that the use of low cost anti-emetics currently available can achieve good symptom control in many cases. Other more expensive antiemetics with little or no evidence of benefit in this setting should only be used in those with refractory nausea or in the context of a clinical trial.

## Abbreviations

CI: Confidence interval
CPGs: Clinical practice guidelines
GIC: Global impression of change
ITT: Intention to treat
N/V: Nausea and vomiting
NRS: Numerical rating scale
PaCCSC: Palliative Care Clinical Studies Collaborative
